# CSF Protein Concentration Shows No Correlation With Brain Volume Measures

**DOI:** 10.3389/fneur.2019.00463

**Published:** 2019-05-03

**Authors:** Alexander Wuschek, Sophia Grahl, Viola Pongratz, Thomas Korn, Jan Kirschke, Claus Zimmer, Bernhard Hemmer, Mark Mühlau

**Affiliations:** ^1^Department of Neurology, Klinikum Rechts der Isar, Technische Universität München, Munich, Germany; ^2^TUM-Neuroimaging Center, Klinikum Rechts der Isar, Technische Universität München, Munich, Germany; ^3^Munich Cluster for Systems Neurology (SyNergy), Munich, Germany; ^4^Department of Experimental Neuroimmunology, Technische Universität München, Munich, Germany; ^5^Department of Neuroradiology, Klinikum Rechts der Isar, Technische Universität München, Munich, Germany

**Keywords:** albumin, brain volume, CSF, protein, MRI

## Abstract

**Background:** CSF protein concentrations vary greatly among individuals. Accounting for brain volume may lower the variance and increase the diagnostic value of CSF protein concentrations.

**Objective:** To determine the relation between CSF protein concentrations and brain volume.

**Methods:** Brain volumes (total intracranial, gray matter, white matter volumes) derived from brain MRI and CSF protein concentrations (total protein, albumin, albumin CSF/serum ratio) of 29 control patients and 497 patients with clinically isolated syndrome or multiple sclerosis were studied.

**Finding:** We found significant positive correlations of CSF protein concentrations with intracranial, gray matter, and white matter volumes. None of the correlations remained significant after correction for age and sex.

**Conclusion:** Accounting for brain volume derived from brain MRI is unlikely to improve the diagnostic value of protein concentrations in CSF.

## Introduction

Cerebrospinal fluid (CSF) analysis is supportive of the diagnosis of many neurological diseases. CSF protein concentrations constitute a mainstay of CSF analysis. Despite age- and sex-dependent cut-offs (1–4), considerable interindividual variance may lower the diagnostic value of CSF protein concentrations. We aimed to reduce variance of CSF protein concentrations and, hence, to increase their diagnostic value by considering brain volumes derived from magnetic resonance imaging (MRI). This idea may not seem practical at first glance but, given latest developments with regard to modern hospital information systems and tools for automated MRI analysis, linking of multiple paraclinical data seems to be in reach even in clinical routine. We reasoned that, since most CSF proteins are both released into CSF (mainly ultrafiltration of blood plasma in the choroid plexus) and retrieved from CSF (drainage into the venous system mostly through arachnoid granulations) in certain circumscribed brain structures, differences in whole brain volumes may not perfectly parallel the net capacity of CSF protein filtration and drainage as only this would lead to independence between brain volumes and CSF protein concentrations. Thus, we studied the relation of CSF protein concentrations (total protein, albumin, and albumin CSF/serum ratio) and brain volumes (total intracranial volume, TIV; gray matter, GM; white matter, WM).

## Methods

The study was approved by the local ethics committee of the medical faculty of the Technical University of Munich. Written informed consent was obtained. We selected patients, who received brain MRI and lumbar puncture, from our in-house database. As a surrogate of a healthy subjects, we firstly reviewed medical records for patients with transient symptoms (e.g., headache) but without a severe or chronic neurological disorder. Based on these strict criteria, we could only include 29 adult patients (age, 31.4 ± 9.1 years; females, 24) as control patients (CP). Given this relatively small number, we secondly selected patients with clinically isolated syndrome (CIS) or multiple sclerosis (MS) aged between 18 and 60 years resulting in a group of 497 patients (age, 35.7 ± 9.4; females, 342; EDSS, 1.45 ± 1.40; CIS, 50.3%; relapsing-remitting MS, 44.1%, primary and secondary progressive MS, each 2.8%). We chose this population, because the suspicion of MS usually prompts performing both cranial MRI and lumbar puncture in clinical setting. Therefore, we could assemble a relatively homogenous and large group of patients with both MRI and CSF data. This approach seemed justified, since total protein levels are normal in 75 percent of MS patients with mild elevation in the remainder (5, 6), whilst levels above 1,000 mg/L are unusual (7). To increase statistical power, we gathered patients with MS and its precursor CIS in one group. Protein levels and albumin CSF/serum ratios were determined by nephelometry (Siemens ProSpec®). As described earlier (8), all brain MRI were acquired with the same protocol on the same 3T scanner (Achieva, Philips, Netherlands). We used a 3D gradient echo T1-weighted sequence (orientation, 170 contiguous sagittal 1 mm slices, reaching down to C4/C5; field of view, 240 × 240 mm; voxel size, 1.0 × 1.0 × 1.0 mm; repetition time (TR), 9 ms; echo time (TE), 4 ms) and a 3D fluid attenuated inversion recovery sequence (orientation, 144 contiguous axial 1.5 mm slices, reaching down to the foramen magnum; field of view, 230 × 185 mm; voxel size, 1.0 × 1.0 × 1.5 mm; TR, 10,000 ms; TE, 140 ms; TI, 2,750 ms). Volumes of GM and WM were obtained from the first segmentation step of the CAT12 toolbox (Version 916, http://dbm.neuro.uni-jena.de), an extension of SPM12 software (Version 6685, http://www.fil.ion.ucl.ac.uk/spm). TIV was estimated by a ‘reverse brain mask method' (9) after lesion filling. For statistical analysis, we used unpaired *t*-tests, simple, and partial correlations in IBM SPSS Statistics for Windows (Version 25.0).

## Results

First, we characterized our data set by testing for well-known associations of the demographic parameters of age and sex with CSF protein concentrations on the one hand and with brain volumes on the other hand. CSF protein concentrations were significantly higher in men than in women [independent-samples *t*-test; protein in mg/L, 624 ± 207 vs. 492 ± 196, *t*_(524)_ = 6.99; albumin in mg/L, 318 ± 122 vs. 233 ± 100, *t*_(524)_ = 8.39; albumin CSF/serum ratio, 6.8 ± 2.4 vs. 5.3 ± 2.4, *t*_(524)_ = 6.41; all *p* < 0.001]. As expected (10), age correlated with CSF protein concentrations (linear correlation; protein, *r* = 0.17; albumin, *r* = 0.15; albumin CSF/serum ratio, *r* = 0.17; all *p* < 0.001). Although CP were significantly younger than CIS/MS patients [independent-samples *t*-test; 31.4 ± 9.1 vs. 35.7 ± 9.4, *t*_(524)_ = 2.38, *p* = 0.018], none of the CSF protein concentrations significantly differed between the two groups [CP vs. CIS/MS; independent-samples *t*-test; protein in mg/L, 489 ± 149 vs. 535 ± 211, *t*_(524)_ = 1.16, *p* = 0.25; albumin in mg/L, 234 ± 83 vs. 260 ± 116, *t*_(524)_ = 1.21, *p* = 0.23; albumin CSF/serum ratio, 5.4 ± 1.9 vs. 5.8 ± 2.5, *t*_(524)_ = 0.96, *p* = 0.34]. With regard to brain volumes, we could replicate well-known associations: men had larger TIV than women [independent-samples *t*-test; 1578 ± 141 vs. 1415 ± 136 ml, *t*_(524)_ = 12.42, *p* < 0.001); GM volume negatively correlated with age (linear correlation, *r* = −0.37, *p* < 0.001].

Given the strong associations of age and sex with both CSF protein concentrations and brain volume measures, we report only results of partial correlation analyses with age and sex as covariates in detail. In the CP group, none of the CSF protein concentrations correlated with brain volume. In the CIS/MS group, we found statistically significant positive correlations of brain volumes with protein CSF concentrations. Yet Pearson correlation coefficients were in the same range as in the CP group suggesting a lack of statistical power to demonstrate these associations in this small group of only 29 subjects. In the CIS/MS group however, none of significant associations between CSF protein concentrations and brain volumes survived correction for TIV, age, and sex (Table 1).

**Table 1:** Correlations of brain volumes with CSF protein concentrations.

**Nuisance variable: TIV, age, sex**	**CSF protein**		**CSF albumin**		**Q-Alb**	
	***r***	***p*-value**	***r***	***p*-value**	***r***	***p*-value**
**Control patients (*****N*** **=** **29)**						
GM	−0.028	0.891	−0.012	0.952	−0.036	0.861
WM	−0.090	0.663	−0.228	0.262	−0.192	0.347
**CIS/MS patients (*****N*** **=** **497)**						
GM	0.061	0.179	0.052	0.254	0.037	0.413
WM	0.021	0.645	0.041	0.359	0.030	0.502

*r, Pearson correlation coefficient; n, sample size; TIV, total intracranial volume; GM, gray matter; WM, white matter; CSF protein, total protein concentration in the cerebrospinal fluid in mg/L; CSF albumin, total albumin concentration in the cerebrospinal fluid in mg/L; Q-Alb, albumin CSF/serum ratio × 10E-3*.

## Discussion

We related brain volumes, derived from high-resolution MRI as available in clinical routine, to CSF protein concentrations. Our data are plausible as we could replicate well-known associations. Men showed higher values of CSF protein concentrations than women. Protein concentrations increased with age. Moreover, we could replicate well-known associations of brain volumes with age and sex. Age and sex are very important clinical parameters; they are available and considered in (almost) every patient in clinical routine and go along with differences in both CSF protein concentration and brain volumes. Therefore, we felt that an association of CSF protein concentration and brain volumes, potentially meaningful in clinical routine, should remain significant after correction for both age and sex.

Accordingly, after having failed to demonstrate a relationship of brain volumes and CSF protein concentrations beyond that explained by age and sex in as many as 526 subjects, we conclude that accounting for individual brain volumes is unlikely to considerably decrease the variability of CSF protein concentrations and, hence, to increase their diagnostic value.

## Ethics Statement

The study was approved by the local ethics committee of the medical faculty of the Technical University of Munich.

## Author Contributions

AW and MM contributed to the conception and design of the study. AW, SG, VP, TK, JK, CZ, BH, and MM participated in the acquisition and analysis of data. AW and MM contributed to drafting the text or preparing the tables.

### Conflict of Interest Statement

The authors declare that the research was conducted in the absence of any commercial or financial relationships that could be construed as a potential conflict of interest.
